# Topical analysis of migration coverage during lockdown in India by mainstream print media

**DOI:** 10.1371/journal.pone.0263787

**Published:** 2022-02-17

**Authors:** Swati Agarwal, Sayantani Sarkar

**Affiliations:** 1 Department of Computer Science and Information System, BITS Pilani Goa Campus, Pilani, Goa, India; 2 Department of Humanities and Social Sciences, BITS Pilani Goa Campus, Pilani, Goa, India; University of Connecticut, UNITED STATES

## Abstract

Implementing countrywide lockdown measures in India, from March 2020 to May 2020 was a major step to deal with the COVID -19 pandemic crisis. The decision of country lockdown adversely affected the urban migrant population, and a large section of them was compelled to move out of the urban areas to their native places. The reverse migration garnered widespread media attention and coverage in electronic as well as print media. The present study focuses on the coverage of the issue by print media using descriptive natural language text mining. The study uses topic modelling, clustering, and sentiment analysis to examine the articles on migration issues during the lockdown period published in two leading English newspapers in India- The Times of India and The Hindu. The sentiment analysis results indicate that the majority of articles have neutral sentiment while very few articles show high negative or positive polarity. Descriptive topic modelling results show that transport, food security, special services, and employment with migration and migrants are the majorly covered topics after employing Bag of Words and TF-IDF models. Clustering is performed to group the article titles based on similar traits using agglomerative hierarchical clustering.

## Introduction

The global Coronavirus pandemic has led to serious structural and functional changes across nations. India encountered its first coronavirus positive case in January 2020 and imposed lockdown and social distancing as steps towards tackling the pandemic (https://www.cnbc.com/2020/01/30/india-confirms-first-case-of-the-coronavirus.html). India declared a nationwide lockdown on 25^*th*^ March 2020 quarantining approximately 1.3 billion people to their homes (https://www.bbc.com/news/world-asia-india-52024239). Initially, the lockdown was announced for a period of 21 days; later it was extended further till May 2020 (https://www.theweek.in/news/india/2020/05/17/lockdown-extended-till-may-31-reports.html). Though the lockdown strictly adhered to the norms of staying at home and restricted mobility, it resulted in a mass movement of the migrant population fleeing the urban areas back to their homes. The sudden lockdown without adequate preparatory measures led to mass closure of the factories, markets and workplaces(https://www.aqs.org.uk/lockdown-chronicle-the-story-of-a-migrant-workers-platform-across-indias-lockdown/). Millions of migrant workers lost their jobs, and being unable to cope with their loss and the related uncertainties, as well as the fear of the virus, they chose to move back to their homes. This set of one of the largest non-clinical COVID-19 issues across the country- the largest domestic migrant crisis since 1947 (https://daily.jstor.org/indias-migration-crisis/). Due to the transport lockdown, large numbers of migrant workers left the cities on foot. The Central government directed the states and union territories to government set up relief camps and shelter homes. The domestic migration drew a lot of media attention, capturing all possible instances of the misery of the migrant workers as well as the relief measures taken. The current article examines and evaluates the topics and opinions of migrant crisis discussion found on press media using text mining. The study uses topic modelling, clustering, and sentiment analysis approaches to mine the news articles and present the patterns of different polarities and issues across migration specific articles. The study also investigates the correlation among different topics and sentiments highlighted by news media during the COVID-19 pandemic in India. The study analyses the language used by print media (here two major daily newspapers) to represent the portrayal of migration issues in the pandemic induced lockdown period in India. The novelty of the study pertains to the methodology to identify the significant patterns in migration news coverage. The following section provides the background of migration issues. The third section discusses the methodology, followed by the section on results and discussion. The final section concludes the article and presents the future directions.

## Background

The story of migration emanates from the prevailing social and economic inequalities across the country [1]. As pointed out by Sengupta and Jha [2], “Social and economic inequalities in India follow the contours of caste, gender, tribe, religion, and regional divisions.” Despite the number of policies and programmes implemented to address the issue, the reliance on the informal sector for livelihood persists [3]. The prevailing social gaps and the limited opportunities in the impoverished areas drive people to move out of their homes to prosperous regions. According to the 2009 World Bank report, [4], in the 1990s, around three million people migrated from Bihar and Uttar Pradesh to Maharashtra and Punjab, primarily for work. A similar flow is evident between the rural and urban areas. According to Census 2011, there are 78.2 million rural-urban migrants in India, and work is the fourth major driver of migration to the urban areas, others being migration due to marriage, with household and after birth. Lack of technical skill serves as a major factor for limiting the scopes of employability of the migrants, and hence they end up in the unorganised informal sector [5]. According to the 2017–18 labour force survey, there are 415 million informal workers in India, making up 90 per cent of the total workforce, and 28 million rural-to-urban workers including small farmers, labourers, weavers and artisans, construction labourers and tradesmen, domestic workers, manufacturing workers, street vendors, transport sector and rag pickers [6].

The onset of the pandemic proved the fact that these unorganised informal migrants remained the most vulnerable community to the greater economic shocks [7]. They are exposed to higher risks of misery and fatality due to their loss of jobs, dislocation, loss of income, loss of home and livelihood, and due to their invisibility from the policies perspectives of the government [8]. Global statistics state that the lockdown has impacted 81 per cent of the working force across the globe, and the worst hit are the informal workers due to the lack of job security(https://bit.ly/39mNdC0) [9]. As pointed out by Sengupta and Jha [2], presently 400 million workers in the informal economy, constituting 90 per cent of India’s workforce, are at risk of falling deeper into poverty. India witnessed the affliction of migrant workers from the very beginning of the lockdown. The sudden proclamation left millions of workers unprepared to deal with the whole situation. Their sudden reaction was of despair, and within hours they were clogging the major transport terminals across the cities hoping to get back to their respective homes [10]. The lack of adequate and on-time response from the government compelled them to choose to walk home [11]. This is evident of the fact of how the cities and the State disowned and deserted an entire section of the population.

The role of the media in relaying information to the general public cannot be denied. Media garners the social responsibility of providing factual information to the common people and educating them about the happenings around them [12]. Media has played a significant role in relaying necessary information related to the pandemic across the nation. The plight of the migrant workers during the lockdown was extensively captured by the media, both print and broadcast, in comparison to the migration issues prior to the lockdown [13]. Within this vast array of information, the present article focuses on using text analysis techniques to identify the major topics and sentiments from the coverage. This study intends to explore the coverage of the migrant crisis in the country by collecting news articles from the two largest circulated English language newspapers- Times of India(https://timesofindia.indiatimes.com/) and The Hindu(https://www.thehindu.com/). We focus on applying topic modelling, sentiment analysis, and clustering-based approaches to determine the topics, opinion polarity and coherence among the topics discussed during the COVID-19 pandemic. Further, the clustering-based approach aims to identify similarities in media coverage from article titles.

Automated text analysis has gained popularity amongst social scientists and policymakers as it has opened up newer avenues towards an understanding of the research questions and related policies [14, 15]. Keller *et al*. [16] applied LDA topic modelling to study the climate change media coverage in two leading newspapers between 1997- 2016. Dahal *et al*. [17] conducted a similar study on climate-related tweets to analyse the public opinions. They used volume analysis and text mining techniques such as topic modelling and sentiment analysis to examine the tweets. Balasubramanyan *et al*. [18] captured the reactions of different political communities on the same news using topic modelling to understand the extent of political polarisation in the US. In another study, Debnath and Bardhan [19] used LDA based topic modelling to understand the government policies to tackle the Corona pandemic in India and found that the interventions generated nudges using external triggers. In similar lines, Liu *et al*. [20] investigated the role of media and patterns of media-related health communication during the COVID-19 crisis in China and found that the emphasis was laid more on larger society than an individual.

Kang *et al*. [21] analysed news media articles on “mukbang” from ten online newspapers over a time span of six years. They used topical analysis as a method to investigate the position of media towards the growing popularity of mukbang and its association with health habits. In another study, Zamani *et al*. [22] proposed content specific LDA topic modelling technique to identify the domains of COVID-19 specific discourse. Similar methods have also been used to study migration issues. In 2013, Allen and Blinders used widespread British newspapers from 2010- 2012 to understand the patterns in coverage of migrant news [23]. Backfried and Shalunts [24] applied sentiment analysis to text documents from relevant media sources to study the migrant crisis in Europe and indicated the more neutral content being published in traditional media. Vazquez *et al*. [25] used topic modelling to extract the most important issues surrounding the migration crisis in Venezuela using online news articles. There are however relatively few studies on migration using text analysis in Indian context. The present study tries to fill this gap by applying the text analysis to investigate the coverage of migration issues by popular print media during the pandemic in India.

## Methodology

### Data collection

**Fig 1:**
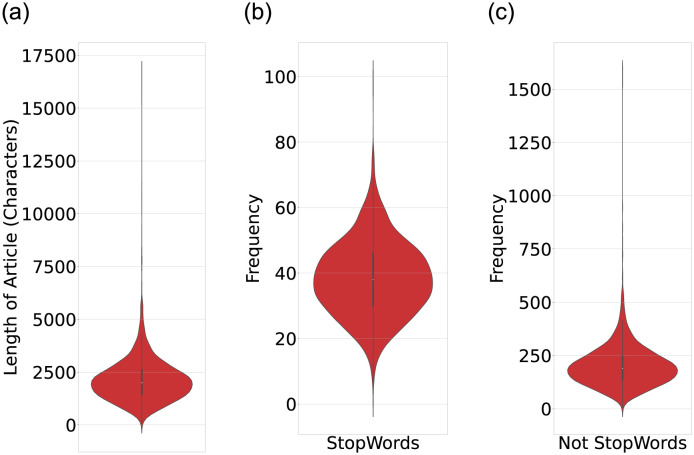
Illustrating the distribution of words across articles.

We conduct our experiments on news articles relevant to domestic migration happening in India during country lockdown amid COVID-19 pandemic. We performed a manual inspection on Indian mainstream printed news media websites and selected The Hindu and Times of India newspapers on the basis of the circulation, popularity and readership. We manually collect the links to these articles to ensure minimum noise (irrelevant articles) in our dataset. We use migrants, migration, lockdown, COVID-19, and pandemic keywords and collect URLs to 2170 unique news articles published in the months of May and June, 2020. The migration related keywords are taken from the existing report on internal migration in India [26]. Further, the COVID-19 pandemic related words are based on the authors’ reading on popular online social media platforms (e.g. hashtags) and news articles. We utilise Newspaper3k (https://pypi.org/project/newspaper3k/) python library to download the news article content from these URLs. We download the article title, authors, body content, article summary, and keywords associated with the articles on these websites. Since all URLs are active and public, Newspaper3k was able to download the content and metadata for all 2170 URLs. Fig 1 shows the violin plot generated for the article length (number of characters), frequency of English stopwords and non-stopwords in the article.

Fig 1 (left) unveils that the articles are of different lengths varying from a very low value approximately up to 17000 characters. The Inter-quartile summary revealed that the articles present in our experimental dataset have a length between 67 to 16, 756 characters. The average length of the articles is 1998 characters, while a majority of the articles are of length 2500 characters (shown by the density distribution and inter-quartile range). These statistics show several outliers with the least and maximum length, but the average value is dominated by the frequency of the articles with a length of 2500 characters. Since the stopwords (common words) help the syntactic structure, their removal does not define the pragmatic and meaning of the sentence. Therefore, we identify stopwords in our articles using NLTK (Natural Language Toolkit) library in Python. We also remove punctuation from text to identify stopwords. The middle violin plot in Fig 1 shows that given the average length of the articles, each article consists of a moderate number of stopwords varying from 0 to 100. The density distribution shows that more than 50% articles contain approximately 40 stopwords. We later perform lemmatisation on non-stopwords, reducing them to their base form based on their part-of-speech tags. For example, in the sentence, “*The migrant workers will have to clear the medical screening at the checkpoints before they are allowed to leave the district*.”, screening (noun) remains same but are and allowed are converted to be and allow, respectively. We compute the frequency of all non-stopwords and illustrate the violin plot in Fig 1 (right). The markers in the inter-quartile range (IQR) unveil that approximately 75% articles have less than 250 uncommon words, whereas upper 25% articles have non-stopwords between 250 to 550. As demonstrated in the left violin plot, the statistics align with non-stopwords revealing articles with more than 500 uncommon words as outliers.

### Sentiment analysis

In this section, we discuss the sentiment analysis performed on news articles. Sentiment analysis is a popular application of computational linguistics to identify and quantify the opinions and effective information in the raw text. The sentiment analysis can be conducted at various granularity levels: document, sentence, aspect (word). Sentiment analysis reveals the opinions and expresses the polarity in the document, i.e., positive, negative, and neutral. We use sentiment analysis to capture the feeling and opinions expressed or disseminated by print media (here two widely circulated english daily newspapers) while covering the migrant issue during the lockdown. We use the article body to calculate the polarity score instead of a specific zone since titles are usually sensitive, whereas a specific paragraph gives the summary or a piece of focused information. To derive the opinion and sentiment in news articles, we use VADER (Valence Aware Dictionary and sEntiment Reasoner) library in Python. VADER utilises a list of lexical features such as the words generally marked as positive or negative according to their sentiment polarity. We used VADER because it provides the gradient of positive, negative, and neutral sentiments for each input. Furthermore, the compound score in VADER calculates the sum of all the lexicon ratings, which have been normalised between -1 (most extreme negative) and +1 (most extreme positive). We discuss the results of sentiment analysis in Results Section.

### Topic modelling

As discussed above, we collected news articles related to migration happened during the COVID-19 pandemic in India. During a manual inspection, we observed that the news articles cover the migration of different social classes and COVID-19 and other topics directly or indirectly related to migration. For example, the other root causes of migration than the pandemic or the after-effects of migration. Since the articles are subjective and contain user-generated content (written by journalists), both formal and informal, we apply natural language processing and data mining techniques to identify the topics discussed in these articles. The topic modelling enables us to organise, search, and understand a vast and diverse range of topics that may be hidden at the time of manual inspection. We use a semantic-based approach because, based on the context, the same word may play different roles in the articles. We applied topic modelling in two phases and discussed them in detail in the below enumerates:

1. Article body: In general, the main body of the article contains the detailed information including the basic introduction to the background of news, concluding remarks, and the references to relevant and previously published news articles. We apply topic modelling on the entire article and identify the topics which are frequently mentioned in our dataset.
2. Summary Text: Since different zones of an article deliver different information (first paragraph, middle paragraph(s), and the last paragraph), we generated the summary of the entire article to reduce the redundancy and retrieve only important information. Text summarisation also helps in understanding the overall context of the article. We use Newspaper3k library to retrieve the summary of the text. It uses TextRank algorithm [27] to rank the sentences in the article and selects top k sentences as the summary. TextRank is an extractive text summarisation technique which generates a summary based on the sentences presented in the input text itself [28]. We use extractive text summarisation because it does not change the words or their syntactic structure in the output.

For each article and summary, we use lemmatised text for identifying topics as discussed in Data Collection section. Topic modelling is an unsupervised learning technique that takes natural language text as an input and identifies the words which best characterise the given set of documents or text. Topic modelling detects the patterns of tokens and phrases within the input sentences/documents and automatically groups these words and expressions to represent the documents. We use topic modelling instead of topic classification since the data is user-generated and unlabelled. Hence, the true classification of topics is unknown. To further evaluate the performance of our topic modelling and validate the results, we use two different techniques for identifying the topics, i.e., Bag-of-words (BoW) and Term Frequency- Inverse Document Frequency (TF-IDF) model.

1. BoW: The Bag-of-words model is one of the simplest techniques in NLP and is widely used method for text processing tasks. A Bag-of-words is a representation of a document that describes the occurrence of words within a document. It primarily consists of 1) A vocabulary of known words. 2) A measure of the presence of known words, i.e., the frequency. The BoW is an unstructured representation which does not contain information about the pragmatic structure of the input text but it rather stores the information about presence or absence of certain known words. For example, in our dataset, terms die, hang, suicide, accident, kill, and blood appear 1163, 2580, 2597, 3570, 3583, and 5002 times, respectively. For each document we create a dictionary reporting the unique words and their frequency referring them to as document vectors. As the vocabulary size increases, the vector representation of documents also increases and mostly leading to a sparse term-document matrix. Initially, the dictionary had 11942 and 7444 words for article and summaries, respectively. The frequency distribution of dictionary terms reveal a long-tail pattern in both summary and article datasets [29]. The long-tail distribution implies that a large number of terms at the far end of the tail have a very low probability of occurrence while there are very few terms that dominates the frequency distribution (usually stopwords). Therefore, we first filter out the tokens that appear in no less than 50 articles and no more than 50%, i.e. 1085 documents. We keep only the first 1000 most frequent tokens for analysis. Thus, the size of the dictionary reduced to 538 (article) and 240 (summary) after filtering process. The filtering ensures the removal of highly frequent words dominating the topic modelling results. For example, COVID-19, lockdown, India, and language function words. Once the vocabulary was implemented, we compute the occurrence of words in example documents and thus we construct the bag of words. This method is also referred to as Word Hashing.
2. TF-IDF: Similar to BoW, we also use the TF-IDF model to identify the topics. There is a limitation to word hashing methods that the highly frequent words start to dominate in the document (e.g. larger score), but may not contain as much “informational content” to the model as rarer but perhaps domain specific words. In the TF-IDF model, we re-scale the frequency of words by how often they appear in all documents, so that the scores for frequent words like “the” that are also frequent across all documents are penalised. We use the TF-IDF approach because we do not remove any stopwords from the document to keep the pragmatic structure intact. Furthermore, the terms which are not present in English language stopwords might be very frequent in the dataset due to domain. For example, COVID. While COVID is an important term but it is obvious at the same time to be present in all documents and have high frequency. The TF-IDF approach uses a term-document matrix which is essentially a multiplication of two matrices, i.e. TF and IDF. Term Frequency (TF) is a scoring of the frequency of the word in the current document. Inverse Document Frequency (IDF) is a scoring of how rare the word is across documents. The scores are a weighted thus not all words are equally as important or interesting. The scores have the effect of highlighting words that are distinct (contain useful information) in a given document. Hence, in TF-IDF based topic modelling, the document vectors are generated based on the TF-IDF scores of each word in the corpus. The cluster formation is followed in the same manner as word hashing. Both BoW and TF-IDF use LDA in background while, TF-IDF additionally use Non-negative Matrix Factorisation (NMF). NMF uses factor analysis method to provide comparatively less weightage to the words with less coherence meeting the goal of TF-IDF.

#### LDA model

**Fig 2:**
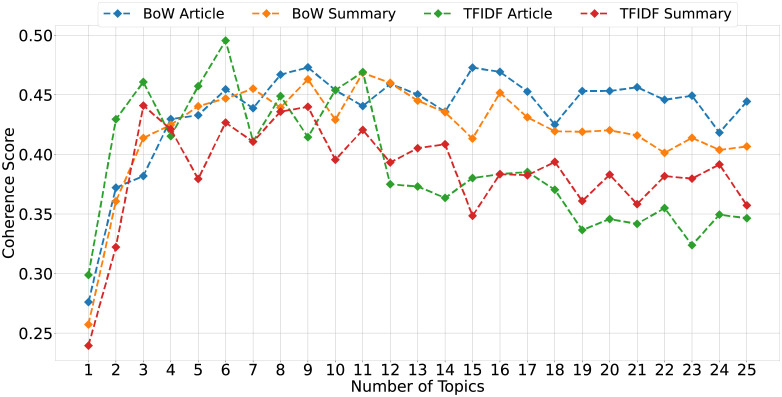
Illustrating the coherence score for different number of topics for bag of words and TF-IDF models employed on article and summary.

Latent Dirichlet Allocation model is a statistical and unsupervised learning model that automatically discovers groups of topics defining a given document. In LDA, the word ‘Latent’ indicates discovering hidden or ‘to-be-found’ topics from the input text. ‘Dirichlet’ is the distribution of words over a fixed set of K topics. ‘Allocation’ is the distribution of topics in documents. LDA assumes that the input document is represented by a distribution of a fixed number of topics, and each topic is a distribution of words. It assigns each word to different topics and determines the topics of the documents by mapping the words present in these documents. The topics are assigned based on conditional probability estimates. The words in certain topics can be selected based on a threshold value for probability or selecting the top *m* words in top *k* topics. We use the Gensim Python library to construct the dictionary and convert it to Bag-of-words and TF-IDF matrix. LDA requires the number of topics as input, and thus, selecting a random value for topics may hamper the quality of topic modelling. We tune the number of topics by optimising the coherence score of topics [30]. For different values of topics *k*, we employ the LDA model on articles and summaries (BoW and TF-IDF) and calculate the coherence score for BoW and TF-IDF vectorizer (Fig 2).

The graph shows that for TF-IDF article and summary corpus, the coherence is maximum for *k* = 6 and *k* = 3, respectively. Though it is not always best to select the *k* with maximum coherence if there are not significant improvement or the scores are fluctuating. For example, BoW summary corpus has highest coherence for *k* = 11 but it does not increase significantly after *k* = 7. Nonetheless, the summary text is relatively smaller than article and thus it is highly likely that the words may overlap across topics. Similarly, for BoW article corpus, the coherence score is same for *k* = 9 and *k* = 15. Further, to yield the clearer results, we tune several parameters of LDA model.

1. **Chunksize** is the number of documents used for training per iteration. We divide our data into five equal parts and thus use chunksize = 434.
2. **Passes** is the number of training iterations through the entire corpus. We use five passes for each input dataset.
3. **Iterations** is the maximum repetition over each document to reach convergence. We run 10 iterations on each document in the corpus. A very low value of iteration may result in no convergence for some documents.

### Clustering

Since topic modelling is an unsupervised technique, we compare the results against standard unsupervised modelling technique, i.e., clustering. The aim of clustering is to divide the population into a number of groups or clusters such that the intra-cluster similarity is higher than the inter-cluster similarity. Thus, the aim is to segregate input documents with similar traits (words in our case) and assign them into clusters. The quality of clustering is dependent on the distance metric used to calculate the similarity between input documents. Similar to topic modelling approach, the collection of documents (corpus) is represented as a term-document matrix where each document can be visualised as a word vector of length *V* where |*V*| is the size of dictionary. Each document can be represented as binary vector (term is present or not), count vectorizer (frequency of a term in the document), or TF-IDF vector (the TF-IDF weight of the term in the doc). As discussed in data collection section, the news article are lengthy documents and have many words repetitive, thus increasing the magnitude of the document in the *V* dimensional space. Therefore instead of news articles, we employ clustering on the news headlines, i.e., the titles collected using NewsPaper3k library. Similar to topic modelling, news headline is represented in the form of TF-IDF vectors. We discard the redundant titles (17) from the data because duplicate data points (or vectors) will end-up in the same cluster and thus increasing the run time of the model. Further, we remove stopwords and non-alphabet strings from the title and use important words for clustering. This results in dimensionality reduction (size of vocabulary and dimensions in vector space). Since each input text is represented as a vector in *V* dimensional space, cosine distance is a suitable metric for computing the distance between two titles. Furthermore, after pre-processing, all empty titles were removed and thus the dataset had 2120 unique titles.

#### Agglomerative hierarchical clustering

**Fig 3:**
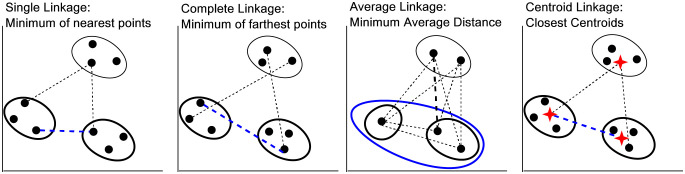
Various linkage metrics for hierarchical clustering. The dashed lines are the linkage between clusters and highlighted edge shows the optimal linkage for clustering.

We use agglomerative hierarchical clustering (AHC) algorithm which is a bottom-up tree based approach [31]. AHC initially assumes each input data point to be a cluster and merges two points at once based on the minimum pair-wise distance, i.e. *ij*^*th*^ element in distance matrix represents the distance between the *i*^*th*^ and *j*^*th*^ cluster. This matrix is updated for each iteration, where elements are updated by pairwise joining until there is one cluster consisting of all data points. The AHC results in a tree-like structure known as dendrogram, displaying the underlying merging process. The tree can be cut at any level based on the number of desired clusters. In addition to a standard distance metric (cosine), hierarchical clustering uses four primary linkage metrics to group the data points (clusters after first iteration), i.e., single, complete, average, and centroid linkage [32]. Single linkage distance groups the data points that are closest (minimum pairwise cosine distance) to each other. Complete linkage groups the cluster points that are least farther (minimum of maximum distance) from each other. Centroid linkage groups the cluster points whose centroids are closest to each other. Average linkage computes the average of *m* × *n* distances calculated between each of *m* and *n* data points of cluster *i* and *j*, respectively. The above four linkage methods have same result for first iteration where each cluster has exactly one data point. Fig 3 demonstrate the distance calculation and criterion for all linkage methods. The linkage method is illustrated for *k*^*th*^ iteration where *k* > 1.

## Results and discussion

### Results of sentiment analysis

**Fig 4:**
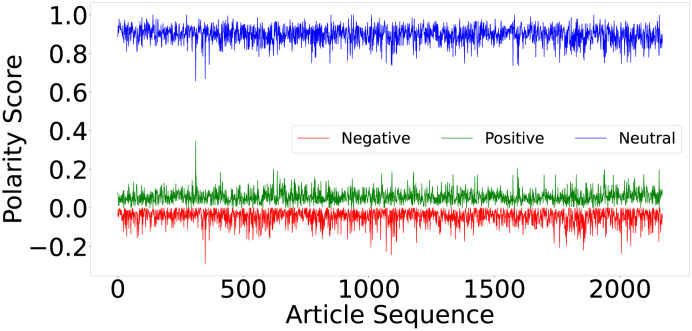
Illustrating the variation in sentiment scores for articles. The positive, negative, and neutral sentiments for each article reveals the ratio of polarities within an article.

**Fig 5:**
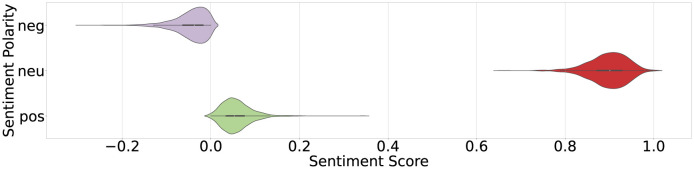
Illustrating the violin plot for distribution of sentiment scores across experimental dataset. The width of the plot at each instance shows the density estimate of articles having a polarity score. In addition to density and distribution, violin plot also shows the inter-quartile summaries of sentiment scores.

Fig 4 shows the polarity score of each article in the dataset. As discussed earlier, VADER returns the likelihood of positive, negative, and neutral polarity for each input text. The spikes in the score show that majority of articles have neutral sentiment. While a few sentences show positive sentiment, other sentences in the same article shows negative sentiment. Hence, overall sentiment of the article turns out to be neutral. Further, the results shown in Fig 5 reveal that even if the articles indicate positive or negative polarity (a few peaks and pits), the confidence/sentiment scores are weak, i.e., closer to ±0.25. The IQR summary shows that 2^*nd*^ quartile (50%) value of the articles is 0.06 which is similar to 0. However, some articles show peaks in positive or negative sentiments. As shown by the graph, 0.34 is the highest positive polarity present only in a few articles. The articles consisting of scores between 0.15 and 0.20 are marked as outliers showing weak polarity.

Similar to positive sentiment, all articles have negative sentiment score between 0 to 0.29 while the median score is 0.05. All articles with sentiment score above 0.15 are marked as outliers. The line chart shows that only one article has the highest positive score (yet below 0.5) the highest peak is visible for one article only. In addition to positive and negative opinions, Fig 4 shows the variation in polarity score for neutral sentiment. The graph in Fig 5 reveals that majority of the articles have high confidence/polarity score for neutral sentiment. IQR summary shows the minimum score of neutral sentiment is 0.66 which is above the baseline 0.50 and thus showing the high confidence. While maximum value is 1.0, the boxplot summary reveals median to be 0.9 which is very high and shows that articles are neutral instead of positive and negative polarity. 3^*rd*^ quartile reveals that not only 50% but 75% of the articles have confidence score of 0.93. In contrast to positive and negative sentiment (IQR = 0.04), neutral sentiment has inter-quartile range of 0.06.

### Results of topic modelling

In this section, we discuss the results of topic modelling acquired from BoW and TF-IDF models applied on both body and summary of the articles. For each model, number of topics were selected based on coherence score as discussed in LDA Model Section. Further, the top 10 terms present in the article mapping with these topics are identified. Tables 1 and 2 illustrate the respective number of topics and their relevant words identified using BoW and TF-IDF models. Each table shows the words mapped from both articles and their summaries.

**Table 1:** Illustrating the words describing various topics identified using BoW model for both article text and summaries.

**Articles**	
Topic 1	case, quarantine, test, covid, centre, health, return, report, number, person
Topic 2	food, help, group, family, water, work, money, woman, time, shelter
Topic 3	court, minister, issue, order, centre, union, congress, party, bench, secretary
Topic 4	village, school, family, year, return, work, child, labourer, shelter, mumbai
Topic 5	travel, transport, border, arrange, jharkhand, place, return, odisha, administration, arrangement
Topic 6	work, employment, return, lakh, scheme, department, minister, number, migration, sector
Topic 7	police, truck, station, road, labourer, vehicle, driver, spot, highway, place
Topic 8	work, labourer, construction, return, demand, industry, city, site, unit, contractor
Topic 9	train, railway, station, passenger, shramik, bihar, board, ticket, reach, official
**Summaries**	
Topic 1	work, district, case, return, covid, labourer, state, construction, number, industry
Topic 2	state, court, health, department, issue, district, order, test, centre, government
Topic 3	home, district, labourer, bihar, place, shelter, city, administration, jharkhand, demand
Topic 4	police, district, labourer, group, truck, home, border, village, road, woman
Topic 5	state, government, minister, home, return, lakh, strand, country, congress, union
Topic 6	train, railway, station, shramik, passenger, board, district, reach, bihar, ticket
Topic 7	food, family, centre, help, water, ration, quarantine, member, home, month

**Table 2:** Illustrating the words describing various topics identified using TF-IDF model for both article text and summaries.

**Articles**	
Topic 1	transfer, account, bank, gandhi, hail, application, certificate, accident, meeting, death
Topic 2	minister, party, congress, industry, employment, work, sector, leader, project, lakh
Topic 3	ration, card, distribute, rice, distribution, packet, food, supply, volunteer, meal
Topic 4	test, case, quarantine, hospital, health, centre, covid, report, sample, person
Topic 5	train, railway, station, passenger, shramik, board, bihar, flight, ticket, travel
Topic 6	police, court, truck, work, family, shelter, labourer, food, village, group
**Summaries**	
Topic 1	government, state, minister, case, court, return, lakh, work, issue, covid
Topic 2	train, station, railway, district, bihar, shramik, passenger, board, border, arrange
Topic 3	police, food, family, village, shelter, help, group, work, truck, labourer

Table 1 shows that among 9 topics for BoW- Article model, even though the topics do not have many overlapping words, it is still difficult to infer the unique topics. This happens to the high frequency of these words in the articles and the high similarity between words themselves. For example, in topic 1, *test*, *return*, *center*, *case*, *quarantine* have some similarity to each other but do not reveal a unique topic. Similarly, while topic 2 is about *providing food and shelter to families*, topic 4 also has the similar terms along with *school*, *village*, *child*, *labourer* which makes it difficult to determine the unique topic in topic 4. Similarly, topic 8 has overlapping words with topic 4. The second part of Table 1 shows the topics and mapped keywords identified for summaries of news articles. These results show article summary are not a good source of determining topics in the articles. The extractive summary consists of the important sentences in the article, thus, all important and relevant words are retrieved. On applying topic modelling, several of these words are selected as candidate words in topics. Since the summary itself is a cohesive and comprehensive representation of the article, these words within an article are treated as very similar. Thus, we see poor topic modelling result (except topic 7) for article summary using BoW model. The results also aligns with the pattern of coherence score for different values of *k* in Fig 2.

Table 2 shows the topics and keywords mapped based on the importance and frequency of the words in the articles. Based on the coherence score, we identified six topics from article data and three topics from summaries. The results show many unique words mapped into topics that were not identified using the BoW model. Further, by manually inspecting these words, they reveal unique topics in a cohesive manner. For example, the first topic is related to the death certificates issued by the hospital, and due to the high similarity in context, banking related words are also identified in topic 1. Topic 2, however, highlights the employment and workers’ conditions in India due to COVID-19. The words such as *lakh*, *project*, *leader*, and *sector* are present due to the workforce getting affected and losing their jobs amid lockdown. Interestingly, the third topic covers the terms which are not identified by the BoW model. Topic 3 highlights the mention of food supplies, but unlike the BoW model, it also identifies that *rice and food were distributed to people who are below the poverty line based on their ration card*. The topic also reveals that *many support groups and citizens volunteered for the meal distribution*. Topic 5 covers the words related to transportation facilities provided to the citizens to return to their homes. The presence of the term *Bihar* validates that maximum labourers were returned to their home in Bihar state. The topic reveals that *special shramik (labourer) trains were started for people to travel back Bihar*. Topic 4 and 6 have overlapping terms with other topics but do not reveal a significant topic or context in the articles. The results show that four out of six topics are tightly coupled groups where terms are highly relevant to each other.

On the other hand, the TF-IDF results for summaries do not reveal meaningful information other than the *food, shelter, and transport facilities*. Due to high frequency within the article but low frequency across corpus, earlier common terms in articles become the important words in summaries. Therefore, the topics are very similar to those acquired from the BoW model, while some similarities are also shown with the TF-IDF model employed on articles. For example, *shramik train* and *shelter* are some of the words identified in the second topic.

**Fig 6:**
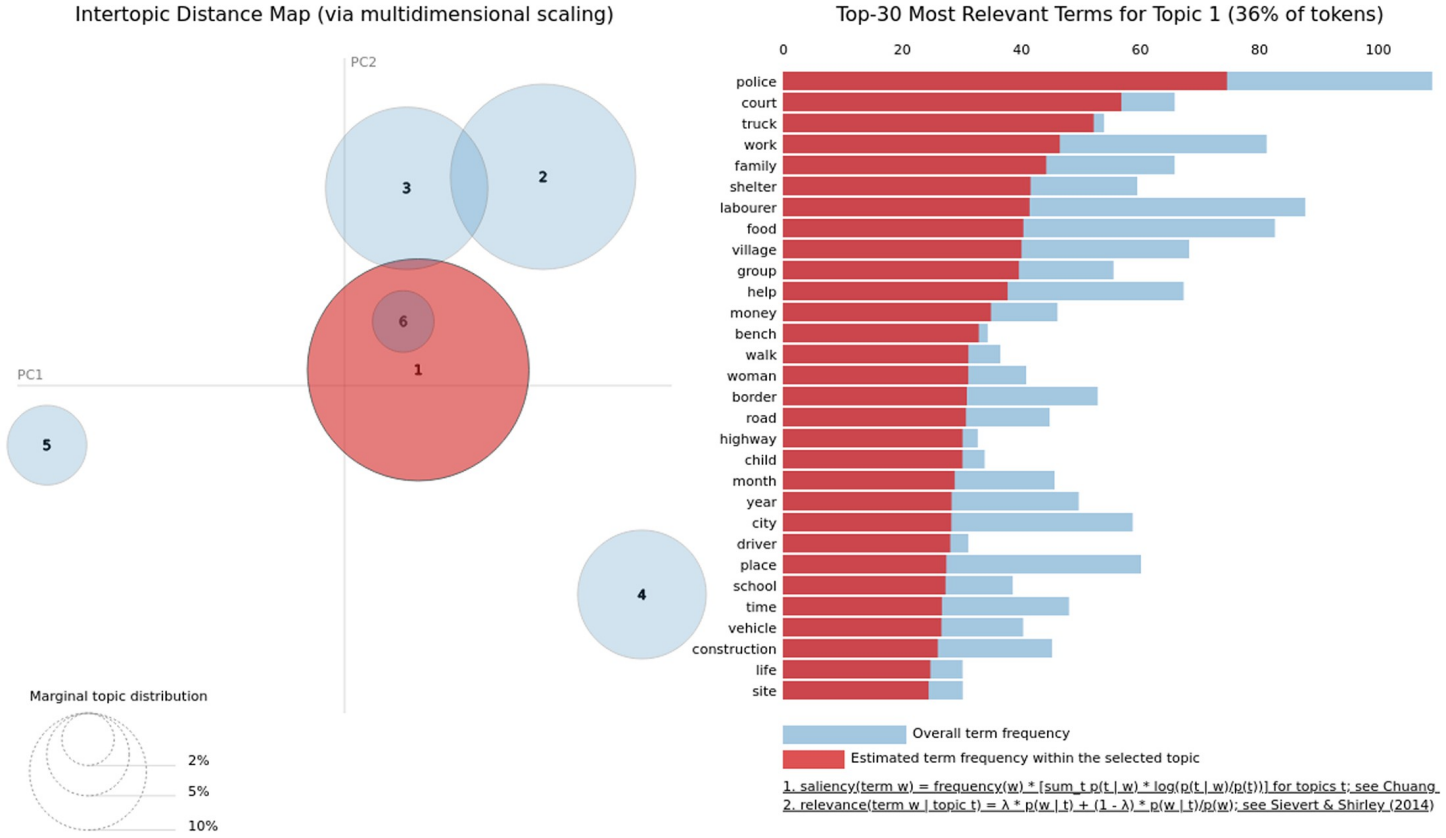
Illustrating the top 30 salient terms present in first topic identified using TF-IDF model employed on articles. The bubble chart visualises the overlap in various topics plotted in a two dimensional (components) space.

We visualised these topics using an interactive model provided by pyLDAvis python library [33]. The model takes topic-term distributions, document-topic distributions, and basic information about the corpus, which the model was trained on as input. Later, we visualised the top 30 terms determining the topic. Due to the space limit, we only presented the illustration for one topic for the article and summary. The graph on the left side represents the bubble/circle chart of marginal topic distribution. The radius of the bubble represents the importance of each topic over the entire collection of articles. The distance between two bubbles (their centroids) represents the similarity between topics. For each (highlighted) topic, the histogram on the right side represents the top 30 most relevant terms present in the topic. Fig 6 shows that topic 6 has all terms overlapping with topic 1 where topic 1 covers the 36% of the tokens of the corpus. Similarly, topics 2 and 3 have a few common words. Note that the sequence of topics in PyLDAvis could be different than the LDA model discussed earlier due to the underlying structure of the algorithms. However, the characteristics of the identified topics show similar behaviour. The bubble chart also reveals that topics 4 and 5 consists of terms that are semantically different from each other. At the same time, they both have terms different from topic 2.

**Fig 7:**
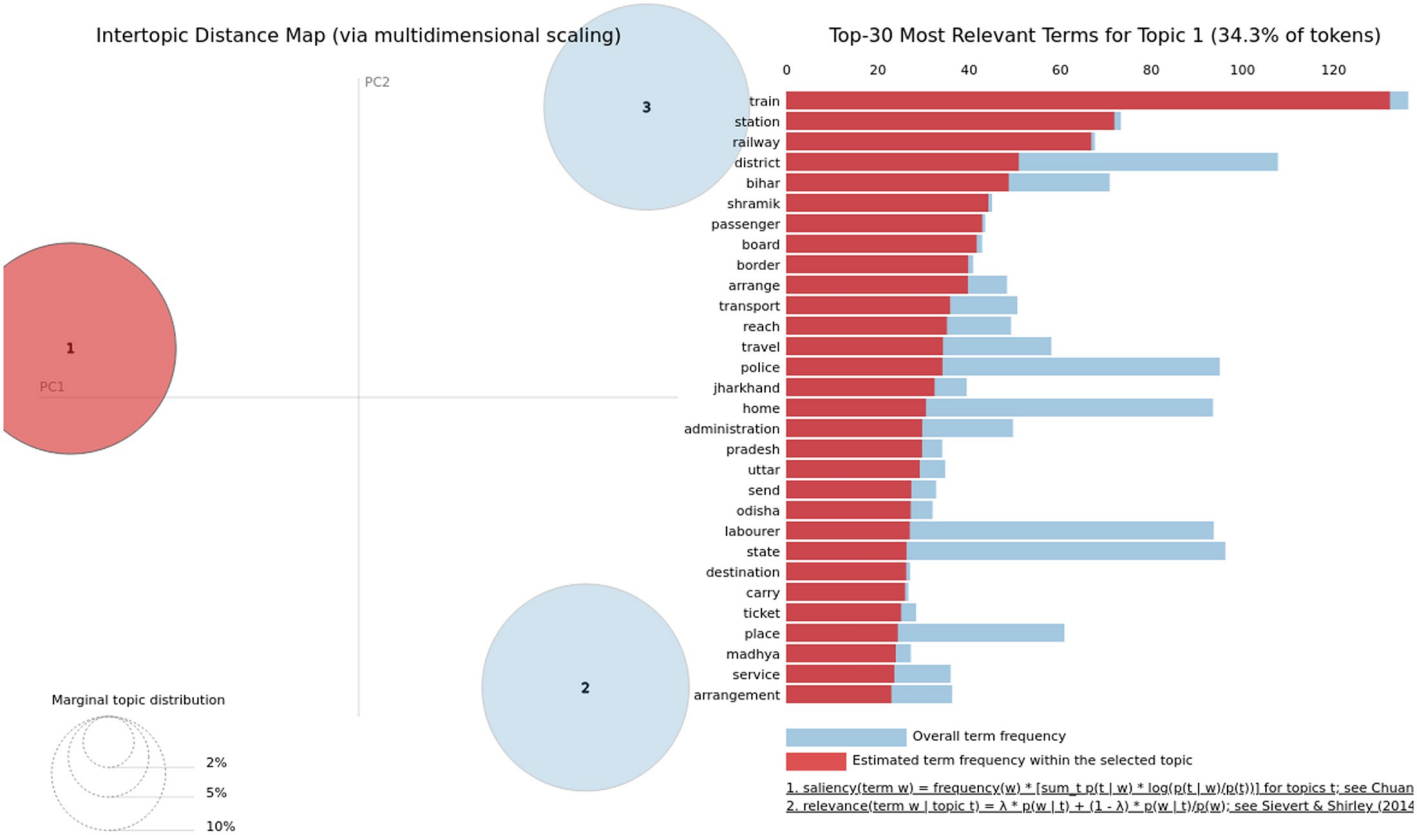
Illustrating the top 30 salient terms present in first topic identified using TF-IDF model employed on articles’ summaries. The bubble chart visualises the overlap in various topics plotted in a two dimensional (components) space.

Fig 7 shows that unlike main body content of articles, all three topics have terms that are semantically different from the terms present in other topics. Interestingly, while top 10 terms presented in Table 2 do not reveal much coherence among the words, the top 30 salient terms in the bar-chart reveal these relations. For TF-IDF vectorization of summary data, topic 1 focuses on the *transportation mediums* and thus contains terms such as *train*, *board*, *shramik*, *travel*, *home*, *send*, *ticket*, and the states’ names where people transported. Based on our inspection, we found that topic 3 covers the keywords related to *food*, *shelter*, *ration*, *money*, *support camps*, *children*, and *labourer*. However, topic 3 contains terms from diverse categories that covers the similar topics as 1 and 3 but contain non-overlapping terms.

Based on our results, we infer that summary of the articles is not a suitable data source to determine the topics. While each and every sentence in the summary is important, they may not be aligned with each other in a semantic manner and thus do not contain sufficient words to represent the document.

### Results of clustering

As discussed previously, the news headlines (titles) are grouped using agglomerative hierarchical clustering algorithm. It first creates a square matrix based on the cosine distance between all pairs of titles then uses complete linkage mechanism to group titles. Unlike conventional distance metrics where clusters have elements that are closest to each other, complete linkage groups the elements that are least far from each other (also demonstrated previously in Fig 3). Thus, the titles within clusters have minimum complete linkage value and are least dissimilar from each other. Further, titles of two different clusters are more far from each other compared to the elements within their cluster. AHC results a tree-based structure i.e. dendrogram of clusters. X axis represents cluster points (initially individual objects at leaf level) and Y axis (height of the tree) represents the complete linkage score between clusters. The dataset has 2120 titles (data points), two or more of which are merged after each iteration.

**Fig 8:**
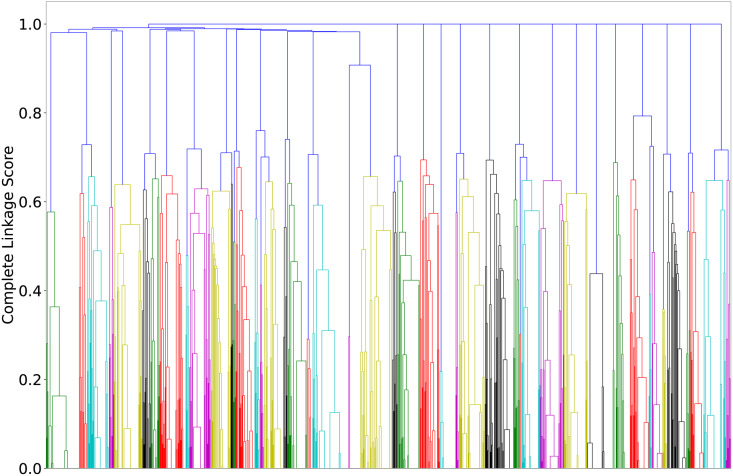
Illustrating the full dendrogram result of agglomerative hierarchical clustering employed on news headlines.

**Fig 9:**
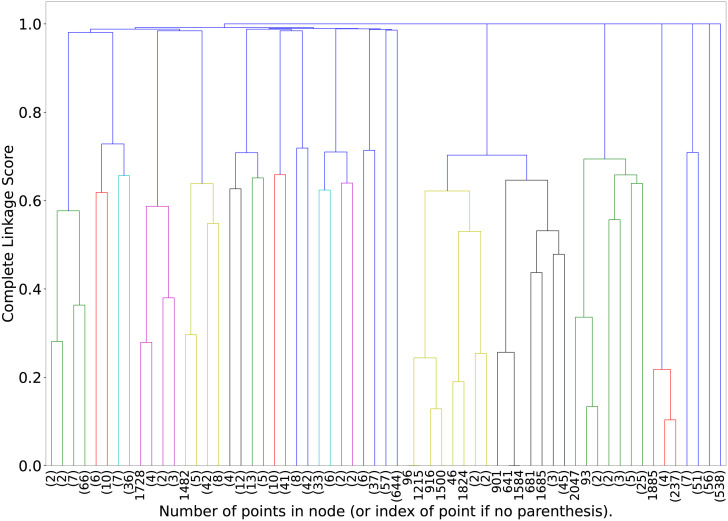
Illustrating the partial dendrogram obtained from agglomerative hierarchical clustering and representing different clusters upto 5 levels from root.

As evident in Fig 8, the number of data points are huge and thus difficult to visualise or interpret in the dendrogram. Thus, we display the dendrogram up to five levels from the root in Fig 9. The X-axis now represents the individual data points as well the cluster with more than one point. The individual values represent the title index in the experimental dataset (maximum 2120) whereas, the values in the parentheses represent the number of data points. For example, the first dendrogram merges two cluster points (2) and (2), which means that two clusters, each having two unique data points, are grouped in one cluster. Furthermore, in hierarchical clustering, each data point is a part of all its ancestor clusters; therefore, it is counted only once when reporting the number of data points at a higher level cluster. Similarly, 9^*th*^ and 10^*th*^ entries on X-axis represent that title index number 11482 is grouped with another cluster of 5 titles which could have been created after grouping five different titles at multiple levels (maximum four levels). The dendrogram shows that at the sixth level from the root, there are 61 clusters consisting of 16 singleton clusters. It further reveals that these 16 titles are very dissimilar to the rest of the titles. For example, index 1728 has only three words after pre-processing, i.e. “Moving migrants non-transparent”. Similarly, index 1482 has terms “kozhikode corporation come bylaw employing migrant”. These words represent that they may not be very similar to other titles related to COVID-19 or migration in India.

We manually go one level down and observe the individual headlines clustered in level six and identify the words present in the titles. We find index 1239, which got clustered at level 6 with four other data points, and it had terms “helping hand nagaland migrant”. Similarly, index 1489 has terms “state cong offer pay migrant rail travel” and grouped with another cluster of two data points at level six. Due to high dimensionality (number of words), it is infeasible to display the cluster results in a two-dimensional space. However, the hierarchical clustering gives us leverage to analyse the results at each iteration and merging process. Furthermore, the tree can be pruned based on the desired number of clusters without relying on initial centroids and outliers. The data points (titles) that are not a part of any cluster at a higher level of dendrogram can be considered outliers since they are the maximum farthest from other titles.

## Conclusions and future directions

Newspapers play a pivotal role in relaying the migration discourse in India to the readers and influence their perception of the issue. The portrayal of migration issues in the form of news articles, columns, and photographs keeps the issue’s vibrancy alive for the readers. Unlike earlier, the pandemic induced reverse migration in India has invited wider coverage by all forms of media platforms, including the newspapers. The present study focuses on analysing the language used by newspapers, using text mining techniques in portraying the issues of the migrants during the aforementioned lockdown period. The study uses sentiment analysis, topic modelling and cluster analysis to examine the articles on migration during lockdown due to the pandemic in two widely circulated English daily newspapers in India- The Times of India and The Hindu. The study pertains to describing the findings more than critically analysing the debate on migration as portrayed in the selected newspapers.

The findings of sentiment analysis indicate that the majority of articles convey neutral sentiment. This can be attributed to the fact that the sentiments get distributed between positive and negative within the articles, making it neutral in nature. The results of the topic analysis indicate that the employed LDA model gives more cohesive results with TF-IDF representation than BoW. The associated topics like Food distribution, Transportation, Death certificates by hospitals, Shramik (labour) special trains, and Shelter facilities were clearly identified besides migration and migrants. Furthermore, the summary of an article is not a suitable data source for topic modelling. While the sentences in summary are important but they are not always cohesive or aligned. The study further performs clustering to segregate the article titles based on similar traits using agglomerative hierarchical clustering. Based on the dendrogram, we conclude that AHC is a suitable clustering technique to generate clusters of news headlines. Further, the outliers can be efficiently identified for different number of clusters or different levels of tree pruning.

The limitations of the study pertain to the fact that the two selected daily newspapers are published in the English language. The usage of a specific language style may influence the captured sentiment and the topics revealed in the topic modelling results. A further analysis of the same issue portrayed in other newspapers, specifically the regionally circulated newspapers in local languages, will help uncover diverse topics and sentiments centred on the migrants during the pandemic. Also, the nature of the newspapers also influences the subjects covered and therefore a more wider selection of the newspapers is needed to understand the position of news media in the migration affair during the pandemic. Further, the analysis of the articles covered only during the lockdown may present a very limited view of the overall migrant issues in the country. The dynamics can be different prior to and after the lockdown ended. Despite these limitations, the study significantly contributes towards understanding the position of news media towards the migration issue during pandemic in India. The study, therefore, opens up future scopes for further analysis in the migration related issues in India using the same method.
